# Lessons Learned from a Case of Abdominal Aortic Aneurysm Accompanied by Unstable Coagulopathy

**DOI:** 10.1155/2012/265860

**Published:** 2012-08-09

**Authors:** Katsuyuki Hoshina, Makoto Kaneko, Akihiro Hosaka, Hiroyuki Okamoto, Kunihiro Shigematsu, Tetsuro Miyata

**Affiliations:** ^1^Division of Vascular Surgery, Department of Surgery, The University of Tokyo, 7-3-1, Hongo, Bunkyo-ku, Tokyo 113-8655, Japan; ^2^Department of Surgery, The University of Tokyo, 7-3-1, Hongo, Bunkyo-ku, Tokyo 113-8655, Japan

## Abstract

Preoperative examination for abdominal aortic aneurysms (AAAs) occasionally reveals an abnormal decrease in coagulation factors and thrombocytopenia, fulfilling the criteria for disseminated intravascular coagulation (DIC). Treatment of the underlying disorder is indispensable for alleviating DIC. We report an uncommon case of a patient with AAA and DIC who showed prolonged thrombocytopenia despite successful treatment of AAA and temporary recovery of coagulation factors. A 70-year-old man presented with AAA and shaggy aorta accompanied by DIC and underwent aneurysmectomy. Combined preoperative use of nafamostat mesilate and recombinant human soluble thrombomodulin was effective in controlling DIC. Although recovery of coagulation factors was observed after surgery, the thrombocytopenia continued throughout the postoperative course and was refractory to platelet transfusion. Because HPA antibody and PA-IgG were present, a trial administration of **γ**-globulin was performed; this resulted in rapid improvement of thrombocytopenia. Although DIC recurred again 2 weeks thereafter, coagulation factors subsequently recovered without any medication.

## 1. Introduction

 Preoperative examination for aortic aneurysms or aortic dissections occasionally reveals abnormal coagulatory conditions that fulfill the criteria for disseminated intravascular coagulation (DIC) [1–4]. Some patients show a clinically apparent bleeding tendency that can lead to massive perioperative bleeding and increase in morbidity and mortality. Although treatment of the underlying disorder alleviates DIC in some cases [3–5], we report an uncommon case of a patient with abdominal aortic aneurysm (AAA) and DIC who showed prolonged thrombocytopenia, recurrence of DIC, and only temporary recovery of coagulation factors despite of successful aneurysm repair.

## 2. Case Report

**Figure 1:**
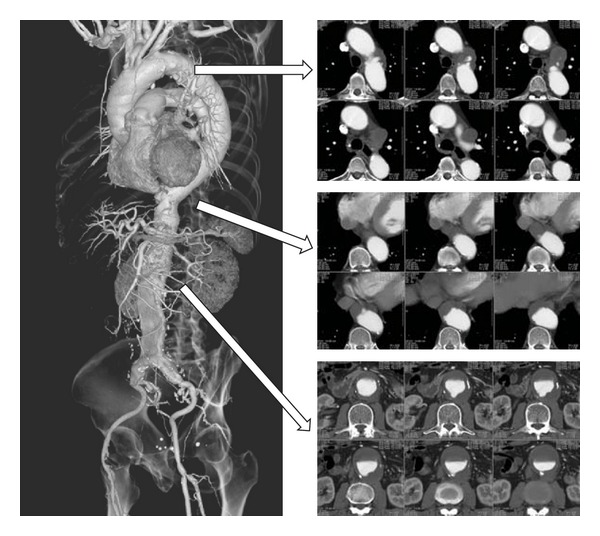
Contrast CT revealed an abdominal aortic aneurysm (anteroposterior diameter, 55 mm) with a partially protruded posterior wall, a saccular thoracic arch aneurysm, and an atheroma-rich “shaggy aorta”.

 A 70-year-old man presented at another hospital in 2009 with general fatigue. Laboratory data revealed a decreased platelet count (2.2 × 10^4^/*μ*L). Additional investigation with computed tomography revealed an AAA with an anteroposterior diameter of 47 mm and a saccular thoracic arch aneurysm measuring 32 × 16 mm, along with an atheroma-rich aorta (“shaggy aorta”). The man had a history of cutaneous purpura 2 years previously, but he had refused to undergo a bone marrow aspiration biopsy. The coronary angiogram also revealed poor cardiac function with an ejection fraction of 35% and severe coronary stenosis. He was followed up until the diameter of the AAA enlarged to 55 mm with a protrusion of the dorsal wall in 2011 (Figure 1). 

Laboratory data showed thrombocytopenia (platelet count, 3.5 × 10^4^/*μ*L), abnormal coagulation factors (fibrinogen, 118 mg/dL; fibrin/fibrinogen degradation products (FDP), 126 *μ*g/mL; D-dimer, 58.2 *μ*g/mL; thrombin-antithrombin III complex (TAT), 25.3 *μ*g/mL; plasmin-*α*2-antiplasmin complex (PIC), 8.5 *μ*g/mL; antithrombin-III (AT-III), 114%); and a high level of brain-type natriuretic peptide (BNP). DIC was diagnosed on the basis of Japanese Ministry Health and Welfare (JMHW) scoring tool [6]. 

**Figure 2:**
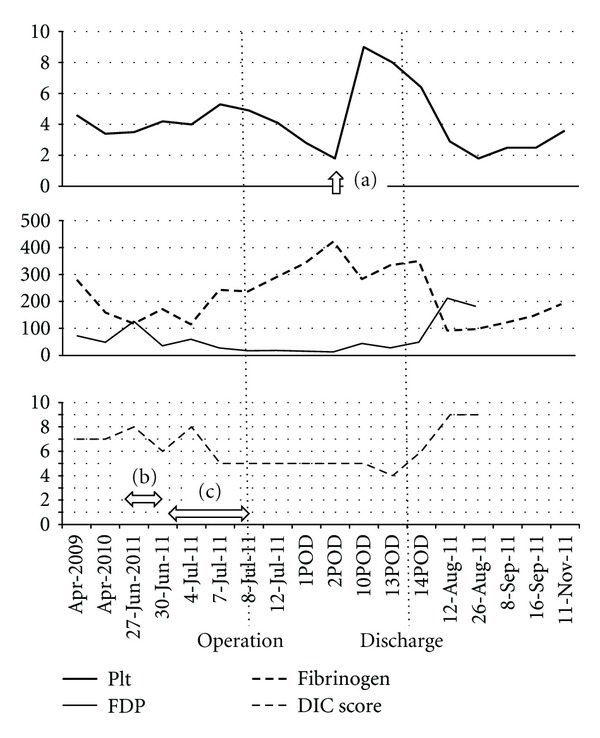
Changes in laboratory data (platelet count and fibrinogen and FDP levels) and DIC score. After administration of *γ*-globulin on POD 3 (a), PTR was alleviated and the platelet count increased. Before the operation, we used UFH for the treatment of DIC but in vain (b). Subsequent use of FUT and rhsTM for 1 week (c) successfully improved the DIC score but did not alleviate the thrombocytopenia.

 Although clinical symptoms of bleeding tendency were not apparent, treatment of DIC was planned before surgery. Initially, continuous infusion of heparin at a dose of 12,000 U/day was administrated for several days. However, the DIC score remained unaltered, and a rapid increase in the APTT value (to 115.0 s) was observed. Continuous administration of nafamostat mesilate (FUT; 60 mg/day) plus recombinant human soluble thrombomodulin (rhsTM: Recomodulin; 19000 U/day) instead of heparin was then performed for 7 days until the day of surgery. The fibrinogen and FDP levels improved from the day after initial administration, thus improving the DIC score; however, the low platelet count remained unchanged (Figure 2).

 Endovascular repair could not be performed because of the large diameter of the proximal aortic neck, and aneurysmectomy and graft replacement were performed. After surgery, the fibrinogen and FDP levels returned to normal without any medication, and bleeding tendency was not apparent. However, the platelet count did not recover despite massive platelet transfusion. We've detected the presence of platelet-associated immunoglobulin G (PA-IgG) and platelet-related antibodies (human platelet antigen antibody (HPA)) against the glycoprotein (GP) IIb/IIIa. Hypothesizing that the antibodies might destroy platelets via an unknown immunological reaction, we attempted *γ*-globulin administration once (1,250 mg) on the third postoperative day (POD), and the platelet level recovered immediately. At 18th POD, when the patient was discharged from our hospital, the platelet count remained at 8.0 × 10^4^/*μ*L and the fibrinogen and FDP levels were still within the normal range. However, 2 weeks after discharge, laboratory data revealed the recurrence of DIC; a decreased platelet count of 1.8 × 10^4^/*μ*L, a lower levels of fibrinogen, and high FDP levels (Figure 2). Because the patient did not show any clinical symptoms of bleeding tendency, he was placed under close observation without any medication. The abnormal value of the coagulation factors is improving gradually over 5 months after the aneurysmectomy. 

## 3. Discussion

 Siebert and Natelson proposed the following criteria for DIC associated with AAA [7]: presence of chronic bleeding disease, consumptive coagulopathy, reversal of laboratory data after aneurysm repair, and maintenance of the data for 3 months. Our patient did not meet these criteria and his platelet level remained low. In addition, remarkable platelet transfusion refractoriness (PTR) was observed. Accelerated platelet activation and immune destruction were main two causes of PTR. Because antibody-mediated immune destruction occurred rapidly within a shorter period [8], an immunologic component was presumed to be the cause of PTR in our case.

 Although a high PA-IgG level is one indication for immune thrombocytopenia (ITP) [9], its level was around the upper border of the normal range in the present case. Thus, in order to exclude a diagnosis of ITP before the operation, we evaluated several additional laboratory tests to further assist in the diagnosis of ITP. We found no evidence of any obvious causes including *Helicobacter pylori* infection, human immunodeficiency virus and hepatitis C virus infection, and antiphospholipid antibodies. HPA assessed by mixed passive hemagglutination test (MPHA) and a routinely using commercial ELISA kit (PAKPLUS, GTI) was clearly detected antiplatelet antibody against the GPIIb/IIIa (HPA-1a/b,3a/b,4a). Since our patient, an elderly male, has no history of transfusion and malignancies, the underlying defects leading to autoantibody production were unclear. We assumed that thrombocytopenia with this patient was partly associated with inducing immune-mediated thrombocytopenia because of the positive test for autoantibody. Accordingly, we administrated intravenous immunoglobulin (IVIg) therapy, which has been reported to be effective in the treatment of ITP [10] and anticipated a favorable effect on HPA-antibody-related reactions; fortunately, rapid albeit temporary alleviation of thrombocytopenia was observed. Good response to treatment with IVIg supported the diagnosis and confirmed the immune nature of the thrombocytopenia. 

Splenectomy for ITP or other hematological diseases without splenomegaly may be controversial given the costs of potent immunity and surgical stress, including surplus operating time and blood loss. Kuwana et al. examined 9 ITP patients who underwent splenectomy and suggested that activation of gpIIb-IIIa-reactive T- and B- cells-induced antiplatelet antibody production in these patients [11]. They also speculated that splenic macrophages act as antigen presenters and activate the gpIIb-IIIa reactive T and B cells, resulting in anti-platelet antibody production [11]. In the present case, if the preoperative DIC state had not improved, we would have considered the simultaneous splenectomy and aneurysmectomy.

 Another enigmatic aspect of the clinical course of the present case was the recurrent DIC. If surgery does not normalize the coagulation parameters, it is necessary to rule out other etiologic factors [7]. The aortic graft itself has been reported to maintain an abnormal coagulation state in some cases [12]. However, our patient showed temporary improvement for a few weeks, which led us to assume that an atheroma from the shaggy aorta or the arch aneurysm was responsible for the recurrence. Pathogenesis of DIC-associated AAA includes exposure of the denuded aortic endothelial surface [1] and a thrombus being a source of local fibrinolysis [13]. Fortunately, our patient did not show any clinically apparent bleeding tendency and DIC gradually alleviated without medical treatment. Recurrence of DIC is possible so long as a massive atheroma thrombus is present on the aorta. 

To attain preoperative control of DIC to facilitate a safe operation on this high-risk patient after a trial of heparin, which proved ineffective and resulted in an extremely high APTT value, we tried combined administration of FUT and rhsTM, which successfully alleviated DIC. One of the reasons we chose rhsTM was that it did not inhibit the initial phase of thrombin generation, and we therefore assumed that the risk of bleeding was lower than that with other anticoagulant drugs [14]. In addition, we expected the drug to have a pleiotropic effect, including anti-inflammatory activity [14]. 

For the future treatment of this high-risk patient, FUT and rhsTM are good treatment options for DIC recurrence and globulin would be helpful for treating aggravation of thrombocytopenia.
